# Book Review: Birds Minds. Cognition and Behaviour of Australian Native Birds

**DOI:** 10.3389/fpsyg.2016.01353

**Published:** 2016-09-07

**Authors:** Giorgio Vallortigara

**Affiliations:** Animal Cognition and Neuroscience Lab (ACN), Centre for Mind/Brain Sciences, University of TrentoRovereto, Italy

**Keywords:** animal cognition, bird cognition, brain asymmetry, comparative cognition, animal intelligence, evolution of birds, evolution of the brain

Passerines, taxonomic group that comprises about two-thirds of the world's existing avian species, originated in the part of Gondwana that today forms Australia (Edwards and Boles, 2002). The ancient supercontinent Gondwana broke up about 180 million years ago, generating the landmasses that we recognize today as Africa, South America, Australia, Antarctica, the Indian subcontinent and the Arabian Peninsula. The book's subtitle makes reference to these major events, which are presented with great accuracy by the author. However, the aim of this book goes well beyond the topic of Australia as one of the cradles of avian evolution. More generally, and interesting not only for comparative psychologists and neuroscientists but also for the general public, this is a book on avian brains and intelligence. I suspect that, in a sense, Australian birds are an excuse for the author, an eminent animal behavior researcher who has made important contributions to the study of Australian (and non-Australian) animal cognition, to discuss some of the more general and central topics of animal intelligence in a broad perspective. In this sense, I believe the book is of interest to readers of any Hemisphere.

Here I focus on a few central topics that I found particularly interesting from the perspective of a comparative cognitive scientist. At the same time, I strongly recommend this book to readers who are more interested in ecology and evolution, topics that are very enjoyable in this book as well.

Curiously enough, the book of Professor Kaplan came to my desk more or less together with the Olkowicz et al. (2016) paper recently published in *PNAS*. With their coincided arrival, I could not resist connecting this new evidence to the problem of reliance on body/brain ratios, which Professor Kaplan discusses so lucidly in her book. Using the isotropic fractionator method (see e.g., Herculano-Houzel and Lent, 2005), Olkowicz et al. (2016) reported that the brains of parrots and songbirds have higher neuron packing densities than mammalian brains, and in particular, that corvids and parrots have much higher proportions of neurons located in the pallial telencephalon. The fact that birds may achieve primate-like levels of cognitive abilities, even though their brains tend to be much smaller in absolute size, is very well-known among comparative cognitive scientists. Gisela Kaplan provided a variety of examples in her book, with a range of topics comprising abstract concepts, tool use, imitation, communication and several others.

Current-living birds that represent more basal lineages, such as fowls and pigeons (neognathes), or emus (paleognathes), have a smaller telencephalon and lower neuronal densities compared to parrots or corvids. In spite of this, however, emus, fowls and pigeons have neuronal densities in the pallium that are comparable to those of the cortex of primates (Olkowicz et al., 2016). And, again, research in comparative cognition has revealed that the most basic cognitive abilities of these allegedly “humble” birds, such as those concerning space, numbers and object representations, parallel those of non-primate (Versace and Vallortigara, 2015) and primate mammals (Vallortigara, 2004, 2006, 2012). Gisela Kaplan's book is in this regard extremely fair and clear when discussing differences between avian species. The author takes the time to always try to explain differences in terms of behavioral ecology and specific selective pressures from natural history, highlighting how these factors shaped their brains and behaviors rather than using nonsensical statements like “more/less advanced species” or “less evolved nervous systems” that sometimes inflate cognitive literature.

The book covers with competence the most traditional topics of avian intelligence, including nest building, mimicry, tool use, imitation, play, social, and vocal learning but also more neglected topics, such as brain and behavioral asymmetries. These latter topics in recent years have developed immensely (e.g., MacNeilage et al., 2009; Rogers et al., 2013) and yet, still tend to remain confined to the realm of specialists. Working together with Lesley Rogers, a world-leading authority on animal lateralization, the author of the book provided original contributions concerning the lateralization of behavior in Australian bird species. For example, studying free-ranging wild Australian magpies with her collaborators, Gisela Kaplan found right eye use in approaching a taxidermized model of a predator and left eye use prior to moving away for escaping. Australian magpies were thus the first native Australian birds ever tested in the field for behavioral lateralization!

Another area of research in which Gisela Kaplan presented extremely original personal research, which added exceptional value to the book, is that of pointing. Traditionally, pointing has been investigated and theoretically discussed, sometimes with acrimony, among primatologists and more recently among scholars who study other mammals (dogs, wolves, dolphins, and horses). The obvious importance of pointing lays in it being a key behavior for the development of language (in children) and theory of mind (in humans and perhaps even in non-human species, though this is debated). Gisela Kaplan has provided, however, intriguing evidence of pointing in Australian magpies. These results have since been replicated in reports on other bird species (Pika and Bugnyar, 2011) and therefore, in light of this evidence of a likely precursor in the evolutionarily distant class of birds, a major reconsideration of the phenomenon of human pointing will be needed.

A major merit of this book is that it integrates with competence and clarity neural, ecological and cognitive aspects of the study of birds' intelligence. This unusual accomplishment likely reflects the author's ample research experience and professional competence, ranging from initial training in social sciences and then to the study of animal behavior, with important experiences in both laboratory and field work, as well as professional activities related to several aspects of animal welfare and veterinary medicine. Overall, the book provides a scholarly but also very enjoyable reading on the intelligence of birds, and should thus be a recommended reading even to non-specialists.

## Author contributions

The author confirms being the sole contributor of this work and approved it for publication.

### Conflict of interest statement

The author declares that the research was conducted in the absence of any commercial or financial relationships that could be construed as a potential conflict of interest.
